# WASP: a versatile, web-accessible single cell RNA-Seq processing platform

**DOI:** 10.1186/s12864-021-07469-6

**Published:** 2021-03-18

**Authors:** Andreas Hoek, Katharina Maibach, Ebru Özmen, Ana Ivonne Vazquez-Armendariz, Jan Philipp Mengel, Torsten Hain, Susanne Herold, Alexander Goesmann

**Affiliations:** 1 Bioinformatics and Systems Biology, Justus Liebig University Giessen, 35392 Giessen, Germany; 2 Algorithmic Bioinformatics, Justus Liebig University Giessen, 35392 Giessen, Germany; 3 Department of Internal Medicine II, and Cardio-Pulmonary Institute (CPI), Universities of Giessen and Marburg Lung Center (UGMLC), Member of the German Center for Lung Research (DZL) and The Institute of Lung Health (ILH), 35392 Giessen, Germany; 4 Institute of Medical Microbiology, Justus Liebig University Giessen, 35392 Giessen, Germany; 5 Center for Infection Research (DZIF), Justus-Liebig-University Giessen, Partner Site Giessen-Marburg-Langen, 35392 Giessen, Germany

**Keywords:** Single cell, RNA-Seq, UMI, Barcode, ddSEQ, 10x, Snakemake, R shiny

## Abstract

**Background:**

The technology of single cell RNA sequencing (scRNA-seq) has gained massively in popularity as it allows unprecedented insights into cellular heterogeneity as well as identification and characterization of (sub-)cellular populations. Furthermore, scRNA-seq is almost ubiquitously applicable in medical and biological research. However, these new opportunities are accompanied by additional challenges for researchers regarding data analysis, as advanced technical expertise is required in using bioinformatic software.

**Results:**

Here we present WASP, a software for the processing of Drop-Seq-based scRNA-Seq data. Our software facilitates the initial processing of raw reads generated with the ddSEQ or 10x protocol and generates demultiplexed gene expression matrices including quality metrics. The processing pipeline is realized as a Snakemake workflow, while an R Shiny application is provided for interactive result visualization. WASP supports comprehensive analysis of gene expression matrices, including detection of differentially expressed genes, clustering of cellular populations and interactive graphical visualization of the results. The R Shiny application can be used with gene expression matrices generated by the WASP pipeline, as well as with externally provided data from other sources.

**Conclusions:**

With WASP we provide an intuitive and easy-to-use tool to process and explore scRNA-seq data. To the best of our knowledge, it is currently the only freely available software package that combines pre- and post-processing of ddSEQ- and 10x-based data. Due to its modular design, it is possible to use any gene expression matrix with WASP’s post-processing R Shiny application. To simplify usage, WASP is provided as a Docker container. Alternatively, pre-processing can be accomplished via Conda, and a standalone version for Windows is available for post-processing, requiring only a web browser.

**Supplementary Information:**

The online version contains supplementary material available at 10.1186/s12864-021-07469-6.

## Background

Since its first application in 2009 [1], single cell RNA sequencing (scRNA-seq) has experienced a steep development. One of the main reasons for this is the unprecedented resolution to analyze cellular heterogeneity. This technology allows evaluation of gene expression for each cell in a sample individually, while traditional (“bulk”) RNA-seq only allowed generation of a mean expression profile of all cells in a sample. As a versatile method scRNA-seq can be applied in various fields of research, e.g. in tumor studies, detection and characterization of previously unknown cell types, analysis of cellular differentiation during development, and many more [2]. Over the last decade, several scRNA-seq protocols have been developed that differ in terms of transcript coverage, sensitivity, cost per cell, throughput, and other parameters. Despite the large number of protocols, an increasing number of studies focus on data obtained by applying Drop-Seq-based protocols [3] which process cells by encapsulating them in aqueous droplets. The main benefit of Drop-Seq-based protocols is a high throughput of thousands of individual cells that reduces the cost per cell, allows simultaneous analysis of larger cohorts of cells and increases the chance to identify rare cell types. Furthermore, commercial implementations of the Drop-Seq method have simplified and expanded its scope of application. However, a higher number of cells makes subsequent data analysis a more complex task. While some steps like mapping or feature extraction are performed in a similar way to bulk analyses and might only show different quality results, a major difference to typical bulk RNA-seq analysis is the need to assign each read to its cell of origin based on a barcode sequence added during the Drop-Seq procedure - also called demultiplexing. This step is critical, because an unsuccessful assignment of reads, e.g. due to sequencing errors, could negatively bias the subsequent analysis steps. Also, demultiplexing needs to be algorithmically tailored to the used implementation of Drop-Seq as the structure of the reads for 10x-based data and BioRad ddSEQ-based data is different.

Another important aspect is the processing of unique molecular identifiers (UMI) used in many Drop-Seq-based experiments. These are unique sequences added to each mRNA fragment to reduce the quantitative amplification bias. Like barcode sequences, they also differ depending on the Drop-Seq implementations and have to be checked as well for e.g. sequencing errors to recover as much information as possible for further analysis steps. The result of this pre-processing is usually a gene expression matrix, in which each row represents a gene and each column corresponds to a cell (barcode). Each entry contains the expression level of the gene in the given cell according to the unique UMIs detected. Post-processing analyses of single-cell data differ fundamentally from bulk RNA-seq data since the results for each individual cell have to be validated to remove low quality cells. Furthermore, a typical aim of scRNA-seq analysis is to cluster cells into cell type specific populations. This poses a completely new challenge compared to a bulk RNA-seq analysis. Following data clustering, the results also need to be visualized with sophisticated algorithms particularly suitable for scRNA-seq data like, for example, t-Distributed Stochastic Neighbour Embedding (t-SNE) [4] or Uniform Manifold Approximation and Projection (UMAP) [5].

These specific requirements show that there is a need for software solutions that are specifically tailored to single cell analysis. Consequently, a variety of software tools have been developed offering a wide range of uses. However, there are a number of potential stumbling blocks that may limit their usability in practice. Several post-processing approaches are implemented as web applications, such as ASAP [6], Granatum [7] or alona [8]. Although this simplifies user handling as these tools provide a graphical user interface (GUI), it also means that users need to upload their data to a remote server which might not be suitable for all experimental data due to the size of the data sets or because of data privacy and protection concerns, especially in medical research. Furthermore, to analyze their data users are dependent on the maintenance of such web servers, which in some cases might result in problems regarding the reproducibility of their analyses.

Other tools are command-line-based, such as ddSeeker [9] or zUMIs [10]. However, these tools only take care of demultiplexing of reads or several pre-processing steps, and naturally they require a certain level of command line knowledge which poses a challenge for many scientists in the life sciences. A third category of tools are software packages typically performing post-processing analysis. There are several Python-based packages like scanpy [11] or Scedar [12]. Other packages are R-based, such as scater [13], SingleCellExperiment (https://bioconductor.org/packages/release/bioc/html/SingleCellExperiment.html) or Seurat [14]. These packages combine a variety of sophisticated algorithms and visualizations, their drawback is, however, that they require users to have advanced knowledge in either Python or R programming. As a result, they are not suitable for most wet bench scientists. Additionally, to our knowledge, there is currently no freely available tool that covers the whole process of pre-processing and post-processing for ddSEQ-based data.

To address the current limitations described above, we have developed the **W**eb-**A**ccessible **S**ingle cell RNA-Seq processing **P**latform (WASP). WASP comprises an automated pre-processing pipeline based on Snakemake [15] and interactive websites implemented with R Shiny (https://shiny.rstudio.com) for result visualizations and post-processing. In order to provide intuitive usage, the software uses a GUI whenever applicable to enable scientists to conveniently process and explore their data. WASP includes an automatic workflow with appropriate default settings for inexperienced users while, at the same time, allowing experienced researchers to adjust all settings in the various processing steps to generate processing pipelines tailored to their specific scientific questions.

## Implementation

WASP is separated into two modules for primary data processing and result visualization, which are implemented in Snakemake and R Shiny. Snakemake is a workflow management system which utilizes a Python-based description language. Thanks to the usage of Conda (https://docs.conda.io/en/latest/) environments, Snakemake ensures reproducibility and facilitates scalable execution of analyses adaptable to the used hardware. Shiny is an R package that allows users to visualize results and dynamically re-calculate them after user interaction. Results and graphics are displayed as interactive web pages which can be further modified using HTML, CSS and JavaScript. These features significantly lower the required level of expertise necessary to analyze data sets with WASP.

### Reproducible pre-processing of raw data using Snakemake

**Fig. 1:**
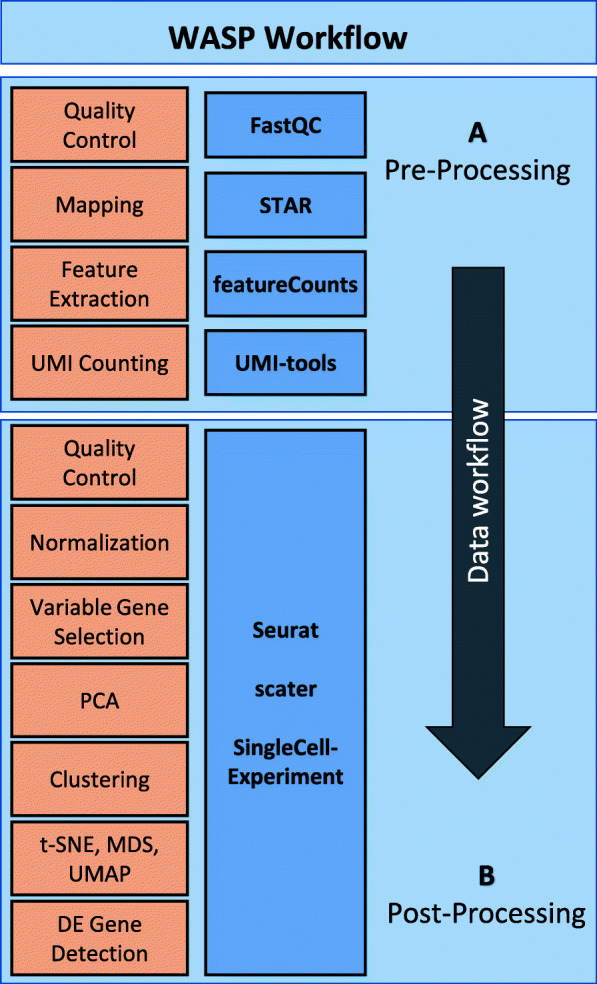
Overview of the analysis steps featured by WASP and the tools used for the individual tasks. **a** Shows the four main pre-processing steps of the Snakemake workflow as orange squares and the tools used for the processing in blue squares. **b** Shows the R Shiny-based steps from the post-processing as orange squares and the single cell specific R packages used during the analysis as blue squares

scRNA-seq data obtained using the BioRad ddSEQ or 10x Genomics protocol is first imported into the pre-processing application. Using a corresponding reference genome and annotation data provided in GTF format, the initial steps of the WASP workflow take care of reference mapping, feature extraction and quantification. This module is implemented as a Snakemake workflow in order to enable reproducibility of results, and users are provided with JSON-based files documenting the quality metrics as well as results of the analysis. The Snakemake workflow utilizes a variety of established tools for the processing including FastQC (https://www.bioinformatics.babraham.ac.uk/projects/fastqc/) to verify the quality of the reads, STAR [16] to map reads to the reference genome, featureCounts [17] to extract reads mapping to exons and UMI-tools [18] to remove duplicate reads originating from the same mRNA fragment (Fig. 1a).

### Inspection of pre-processing results and estimation of valid barcodes

For a user-friendly assessment of the results, a Shiny web application is provided presenting information about each step, e.g. number of extracted barcodes, number of mapped reads/extracted features per barcode or number of UMIs per barcode. Besides the visualization, the Shiny app also addresses the selection of correct barcodes. This is of vital importance because a major challenge in Drop-Seq-based protocols is the verification of the number of barcodes belonging to “real cells”. This problem arises due to the incorporation of free RNA released from broken cells inside the droplets during the cell separation. As a consequence, some droplets contain these free RNAs instead of mRNAs from one whole cell. Free RNAs are processed similarly to mRNAs from a whole cell and appear in the data with a barcode and UMI. However, these reads should be filtered out as they mark false-positive hits and would increase noise for the post-processing and also slow down further analyses. WASP calculates a cutoff containing all barcodes expected to belong to intact cells. For this, a ‘knee plot’ is calculated, by ordering all detected barcodes descending on the x-axis according to the number of UMIs. The y-axis shows the logarithmic number of UMIs identified in each barcode. To determine the cutoff, the first turning point of the plotted curve must be calculated. This is done by ranking the barcodes in descending order by their UMI counts. Subsequently, a score is calculated taking the difference in logarithmic UMI size between each two neighbouring barcodes divided by the difference in their rank to take into account when multiple barcodes share the same rank. Additionally, users can select a minimum of expected cells in the uploaded data to aid the cutoff detection. Finally, the barcode showing the largest difference in this metric is selected as cutoff. However, users can increase or decrease the number of barcodes to be used manually for the following analyses. Ultimately, based on the selected cutoff a gene expression matrix is generated, in which rows represent the genes and columns correspond to the selected cells (barcodes). The entries contain expression levels according to the number of unique UMIs per gene per cell. To enhance reproducibility, users may also download a table containing all parameters of the tools used during the Snakemake analysis as well as the selected number of barcodes. The gene expression matrix can then be transferred to the post-processing application for downstream analysis. Of course, the post-processing can also handle similarly formatted gene expression matrices generated by other pre-processing pipelines.

### User-friendly visualization of results using R shiny

**Fig. 2:**
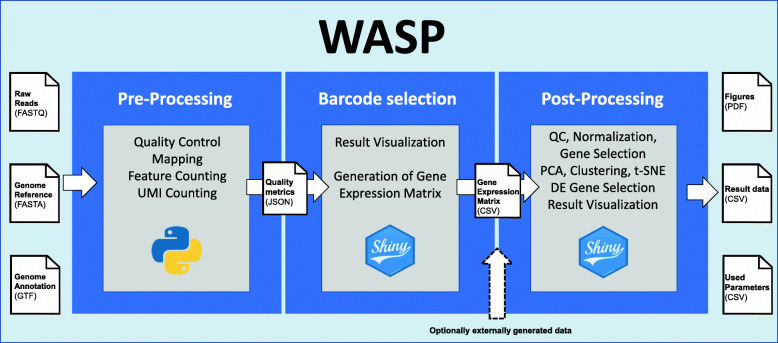
Schematic overview of a WASP analysis. As a first step, users start the Snakemake workflow on a Linux-based system, providing the FASTQ file with the reads and a reference genome with corresponding annotation. The results (quality metrics) of the workflow are then presented in an R Shiny web application which generates a gene expression matrix CSV file containing UMIs per gene and cell. This file or a similar externally generated file are then uploaded to the post-processing Shiny web application for further processing. Post-processing can be performed in an automated or manual mode and presented as a dynamic web page similar to the pre-processing results

The post-processing of the filtered scRNA-seq results is based on an additional R Shiny-based application, which requires input of a gene expression matrix obtained from the pre-processing step (for BioRad ddSEQ and 10x Genomics protocol-based experiments) or obtained from other sources (Fig. 2). After uploading the corresponding data analysis files, the user is offered access to two different types of analysis: an automatic mode or manual analysis. The automatic mode is designed for less experienced users, calculating all analyses in one run with default parameters and storing the results from each step for later visualization. After the analysis, the user can browse the results in interactive web pages. More experienced users can select the manual mode which calculates the analyses stepwise, giving users the opportunity to change parameters between steps. Finally, all results are presented as an interactive web page, similar to the automatic analysis.

Before starting the analysis various filter options can be selected, such as thresholds for the amount of UMIs and genes per cell or the number of transcripts per gene, thereby allowing removal of cells with low quality. WASP provides different means in order to normalize the UMI counts between cells, cluster cells into subpopulations, and also facilitates the identification of differentially expressed genes based on the Seurat R package. This includes selecting highly variable genes to reduce noise in the further analysis and dimensionality reduction based on principal component analysis (PCA). Convenient visualizations such as t-SNE, UMAP and multidimensional scaling (MDS) enable assessment of details such as cluster assignment or expression of specific genes in all cells/clusters. All obtained results are provided as publication-ready high-quality charts in PDF format or as CSV-based data exports for additional processing with external applications such as MS Excel. Additionally, a CSV-based file is generated documenting all used parameters to support reproducible analyses (Fig. 1b).

### Separate processing provides flexibility

The separation of WASP into two modules offers several benefits. One key advantage is that this enables optimal adaptation of the software to the different hardware and software requirements involved in pre-processing and post-processing. Pre-processing includes a mapping step as one of the main processing tasks. Because scRNA-seq data is normally based on eukaryotic organisms, often human or mouse cells, mapping usually requires a higher amount of memory (RAM). Another critical and computationally intensive step is the demultiplexing as every mapped read has to be checked for a valid barcode and UMI sequence. We provide tailored pre-processing routines to extract ddSeq- and 10x-specific barcode and UMI sequences. In order to speed up the demultiplexing, we implemented this step as a parallel process while maintaining reproducibility using Snakemake (Fig. 2). Another aspect is the usage of well-established tools to yield the best possible results from the input data as some of these tools are designed to work on Linux-based systems only. Therefore, the pre-processing significantly benefits from a Linux-based environment with multiple cores and a high amount of RAM such as a compute cluster or workstation.

In contrast to this, post-processing is implemented with R-based scripts and packages, allowing it to be used on other operating systems as well. Also, post-processing generally does not require such a high amount of RAM like the pre-processing and does not benefit as much from a high number of CPU cores, as its calculation is typically performed within minutes on a standard laptop. Furthermore, the post-processing starts with a gene expression matrix making it independent from the protocol used for the scRNA-seq data generation (Fig. 2). Since scRNA-seq is still a relatively new technology, there are some specific problems during post-processing in some data sets. Experienced users, however, might be able to solve these issues and retrieve more information by changing specific parameters.

To fulfill these requirements and to offer users the highest possible flexibility, we have separated the pre-processing and post-processing into two parts. This allows users to easily process ddSeq- and 10x-based reads while benefitting from established tools during the pre-processing. In addition, users are able to use the post-processing on normal computers or even laptops based on Linux or other platforms such as Windows or MacOS. The separation further expands the application of WASP to include scRNA-seq data from protocols such as 10x Genomics. Also, the post-processing enables experienced users to modify parameters in order to extract as much information as possible from challenging data sets.

To allow easy use, both pre-processing and post-processing are available via Docker, eliminating the need to install further dependencies. Moreover, the pre-processing can easily be installed using Conda and the post-processing is available as a standalone version for Windows, requiring only an installed web browser.

## Results and discussion

### Generation of a gene expression matrix for ddSEQ-based data

In order to use the pre-processing pipeline, the user needs to provide a reference genome with an annotation and the raw FASTQ files (Read 1 and Read 2) generated with BioRad’s ddSeq or 10x Genomics protocol. For this, a dedicated directory structure is used, that can be obtained from the git repository (Figure S1). After preparing the input data, the Snakemake pipeline can be started by a single command line, with the two mandatory parameters being the number of cores to be used and the scRNA-seq protocol.

**Fig. 3:**
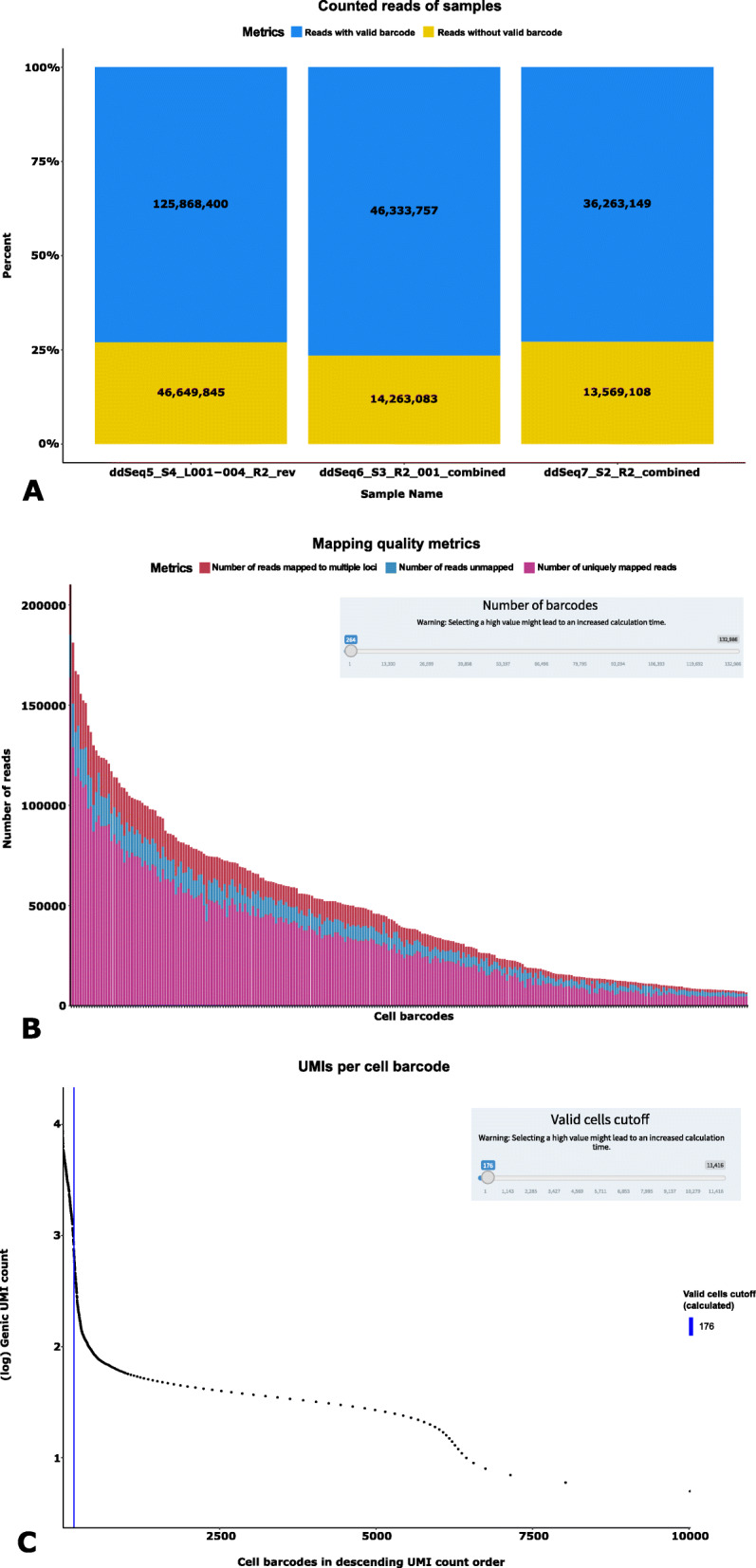
Examples of WASP pre-processing quality metrics. **a** A summary page shows quality metrics about identified barcodes, STAR mapping and featureCounts analyses. **b** Results for each analysis step, e.g. the STAR mapping, are presented in a detailed page as well with interactive selection of metrics (e.g. categories of mapped and unmapped reads) **c** On the last page, users select the number of barcodes to be used for further analysis. By calculating a knee plot, WASP provides users with a suggested number of detected true-positive barcodes

Following the Snakemake run, the result data is visualized using the dedicated Shiny application. At the beginning, the user has to select the “results directory” generated with the Snakemake workflow. Subsequently, a “sample summary” page is presented (Fig. 3a), showing an overview of quality metrics for each processed sample. These quality metrics are separated into three blocks showing the information as a table and a stacked barplot: 1) The first block contains information about the number of reads with and without a valid barcode and the number of extracted barcodes. 2) The next block shows information regarding the STAR mapping results, such as uniquely mapped reads, unassigned reads, etc. 3) The last block shows information about featureCounts results, such as the number of reads assigned to a single feature, reads assigned to multiple features, etc. A menu on the left side of the web pages allows the user to navigate through other result pages:

- FastQC Report:
  This page provides users with the quality control results generated with FastQC. To provide an overview of the sequence quality of the samples, graphics generated with FastQC are shown on this page.
- Mapping Rates (Fig. 3b):
  This page shows the fraction of uniquely mapped reads, unmapped reads, and reads mapped to multiple features for each cell using stacked bar plots. By changing the amount of cells to analyze, the plots are dynamically re-calculated. Additionally, users can browse through the data corresponding to each barcode in tabular form for the mapping statistics of uniquely mapped reads, reads mapped to multiple loci, and unmapped reads.
- Gene Counts:
  Similar to the “Mapping Rates”, this page shows fractions of the read data assigned to a feature or to different categories of unassigned reads. Again, users can dynamically re-calculate the stacked barplot by changing the number of cells to analyze or by selecting the categories of reads to be shown. At the bottom of the page, users can browse identified barcodes in tabular form.
- Valid Cells (Fig. 3c):
  As mentioned before (“Inspection of pre-processing results and estimation of valid barcodes”, Implementation), a major challenge in Drop-Seq-based protocols is the discrimination of barcodes added to free mRNA fragments from barcodes added to mRNA fragments of a whole cell. This page shows the ‘knee plot’ and the automatically computed cutoff. Additionally, pie charts below the knee plot show the distribution of mapped reads and identified genes in the selected barcodes. When the number of valid barcodes or expected number of cells is changed, these plots are updated. Also, users can browse through the barcodes in tabular form to get a detailed view of identified UMIs and genes per barcode.

After selecting a number of barcodes to be used for further analysis, users can simply download the gene expression matrix and a list of used parameters by clicking a button (Figure S2). The gene expression matrix can then be used with the post-processing WASP application for additional analysis.

### Customizable analysis and dynamic visualization of scRNA-seq post-processing

**Fig. 4:**
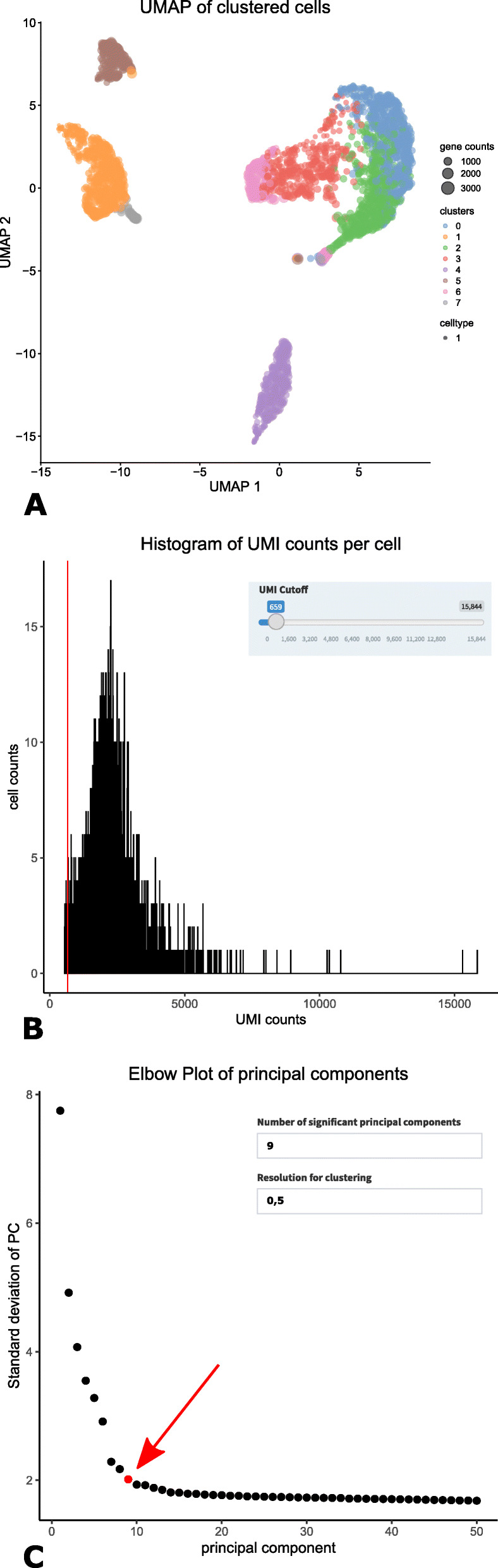
Examples of WASP post-processing analyses. **a** 2D UMAP plot of clustered cells with detailed information about a selected cell. **b** After starting the Shiny web application in manual mode, users have to select a threshold defining below which number of UMI counts a cell is discarded. **c** The elbow plot showing the standard deviation of each principal component and a calculated recommended cutoff (red dot and arrow) for use in the following analyses. Users can select custom values for the clustering resolution and the number of principial components used for the following analysis steps

To make the WASP software also applicable for datasets which were created using a protocol not supported by the pre-processing pipeline, the post-processing was designed as a stand-alone module. The post-processing Shiny application can be started either as a Docker container or as a standalone application on a Windows-based PC. A main advantage of the latter is that it does not require the installation of any additional software or packages, only a standard web browser is needed. After the application is started, the user is provided with a web page similar to the Shiny pre-processing page. This page also contains examples of how the input data has to be structured. As a first step, the user has to upload the gene expression matrix file. Where applicable, it is possible to upload an additional annotation file containing all cell barcodes and their assigned type. This can be useful if data from several experiments are combined to label each cell with its origin. Another option is to add already known cell type information, e.g. if cells have previously been selected from specific tissues. When an annotation file is provided, WASP shows users the added cell information in plots allowing to check for e.g. batch effects. After the upload is completed, users can choose between an automatic and a manual analysis mode. The first mode was integrated to allow less experienced users to gain easy insight into their data. For this mode, we evaluated parameters from previous analyses to select default values suitable for most data sets. In the automatic analysis mode, WASP calculates all analysis steps and presents the graphical results on a new web page. The manual mode however allows users to change parameters before each step. Therefore, WASP calculates the results step-by-step and presents the user only with the results of the current step, such that the user can decide which parameters to use for the next step. To simplify the usage, default parameters will be used if the user continues to the next step without changing the values. Similar to the automatic mode, WASP presents all generated visualizations on one page after all processing steps have been executed. A menu on the left side allows users to select each analysis step and to re-calculate the analysis with new parameters from this step on. Since WASP uses the newest version 3.1 of the Seurat R package internally for many analyses, its workflow is highly similar to the Seurat workflow. In addition to Seurat, WASP uses the R packages ‘SingleCellExperiment‘and ‘scater‘to provide users with additional analyses and information about their data set. For scientists without a certain level of expertise in R programming the usage of the three mentioned packages would be challenging, even more so if they wanted to combine the results of several packages to enhance their analysis results. The R Shiny GUI-based web interface of WASP offers users an easy-to-use tool to perform post-processing scRNA-seq analysis (Fig. 4).

The analysis steps cover a typical scRNA-seq workflow, beginning with quality control to remove low quality cells from the data, e.g. cells with a very low amount of UMIs or detected genes (Fig. 4a). To account for technical noise and focus on biological variability, the data is normalized, scaled, and genes with a high variation are used for dimensionality reduction of the data set via principal component analysis. During the manual workflow, a user has to decide how many of the calculated components will be used in the following analyses. This step is important, as selecting a value too low could negatively affect the results and might cause a loss of important data for subsequent clustering. However, in some cases a too large value could also lead to an increase of noise in the data. Several plots aid the user in this decision, primarily Seurat’s so-called elbow plot, which shows the standard deviation of each principal component. Additional to Seurat, a cutoff is calculated in WASP by fitting a line to the elbow plot curve connecting the points with the highest and lowest standard deviation, identifying the perpendicular line for each point to the connecting line and selecting the point farthest to the connecting line. This cutoff is detected to recommend how many dimensions should be included in the following analyses (Fig. 4b). This calculation is also used to select a cutoff during the automatic workflow. Based on the selected value, cells are clustered according to Seurat’s graph-based clustering algorithm. The clustered cells are shown utilizing a variety of sophisticated visualizations including t-SNE (Fig. 4c) and UMAP. As an additional feature, WASP combines the clustering information with data about the cell size. Finally, genes that are differentially expressed between clusters are identified. During the manual workflow, users are able to select custom *p*- and log_2_foldchange-values for this analysis. WASP includes a variety of visualizations such as t-SNE, UMAP, violin plots, ridge plots, dot plots and heatmaps, allowing users to dynamically check the expression levels of genes between clusters. Users simply need to type in the name of a gene of interest to calculate plots, showing its expression among the different clusters. Furthermore, WASP provides an interactive table containing all genes identified as differentially expressed in a cluster with corresponding *p*-values and log_2_foldchange-values, allowing users also to search genes by name. On the last page, users are provided with all generated visualizations including the option to download these along with the calculated normalization table, the list of marker genes for each cluster and a summary table containing the used parameters to support reproducible analyses.

### Successful processing of ddSEQ and 10x genomics data sets

To assess the performance of WASP, we performed a test run with a ddSEQ-based data set of roughly 283 million reads separated into three samples (ddSEQ5, ddSEQ6, ddSEQ7) originating from *Mus musculus* cells. The raw data is available at the NCBI Short Read Archive (SRA) repository under the SRA accession number SRP149565. For the processing we provided 12 CPU cores of the type Intel Xeon E5–4650 running with 2.70 GHz each. The peak memory usage monitored was 28 GB RAM, and the total processing time was approximately 3.5 h for the previously mentioned steps of FastQC, mapping, feature extraction and UMI counting. Subsequently, results were processed using WASP’s pre-processing Shiny application to select a number of cells to be used for generation of the gene expression matrix. Using this method, WASP automatically selected 346, 399 and 176 valid barcodes for ddSEQ5, ddSEQ6 and ddSEQ7, respectively. We combined these cells to further evaluate the post-processing application.

Subsequently, the resulting gene expression matrix was analyzed with the post-processing module of WASP. This test run was performed with the Windows standalone version of the Shiny app on a laptop with 8 GB RAM and 2 CPU cores (Intel Core i5-4510H) running with 2.90 GHz each. The total processing time of the data for all 935 cells from initial import of the gene expression matrix to generating cluster results including visualizations such as t-SNE and UMAP and differentially expressed gene tables was less than 3 min. The results of this analysis are discussed in detail in Vazquez-Armendariz et al., EMBO J. 2020 [19].

We performed a second test to assess the performance of WASP for 10x Genomics data sets as well. In order to validate the recently implemented pre-processing of 10x barcoded single cell data we’ve used a publicly available data set of roughly 132 million reads combined from two sequencing runs originating from *Ciona robusta* larval brain cells. The raw data set is accessible under the SRA accession number SRX4938158. Using the same hardware as mentioned above in the ddSeq test, the pre-processing had a total run time of approximately 2 h with a peak memory usage of 5.3 GB RAM. Following the run, WASP’s Shiny application detected 2816 valid barcodes. In a second test, we’ve validated WASP’s performance on an externally generated expression matrix based on the 10x protocol. For this test, we used a gene expression matrix containing 2700 peripheral blood mononuclear cells. This data set is publicly available under: https://support.10xgenomics.com/single-cell-gene-expression/datasets/1.1.0/pbmc3k. For this test, we used the same setup as mentioned above with the Windows standalone version. The total processing time was 6 min, which took longer than the ddSEQ data set as the 10x data set is about 2.8 times larger than the ddSEQ test data set. Following the analysis, we compared the results to an exemplary analysis of the same data set on the Seurat homepage. WASP was able to identify similarly composed clusters with the corresponding marker genes.

## Conclusions

Ten years after its development, single cell RNA sequencing is still an emerging technology. Compared to traditional bulk RNA-seq it allows an unprecedented view on cellular heterogeneity and enables researchers to analyze individual gene expression profiles of each cell in a sample individually. These opportunities led to a broad field of applications and a variety of technological modifications. During the last years, scRNA-seq was combined with the Drop-Seq technology to massively increase the throughput of cells while substantially reducing the cost. These improvements come with the cost of a more complex analysis requiring appropriate software solutions. However, a recent study [20] clearly identified a lack of freely available tools supporting the analysis of scRNA-Seq data. With WASP, we provide a comprehensive and user-friendly open-source software solution enabling scientists to process and interpret their scRNA-Seq datasets obtained with the ddSEQ and 10x protocol. To the best of our knowledge, it is currently the only non-commercial software package capable of performing the combined pre- and post-processing of ddSEQ-based scRNA-seq data. Due to its separation into two modules, the post-processing application can be applied to scRNA-seq data from other protocols as well. One of the main priorities during the development of WASP was to keep its usage as simple as possible and to minimize the installation and maintenance effort. Thus, WASP is offered as an easy-to-use Docker Container, it utilizes a standardized Conda environment and the post-processing module is executable as a Windows standalone application. WASP can be used on-premises and does not require uploading potentially sensitive data to an external server. Thanks to its user-friendly web-based GUI and its automatic analysis mode, it allows experimental scientists to quickly take a first look at their data. Additionally, parameters can be adjusted in the manual post-processing mode to optimize the analysis for experienced users. Finally, WASP produces publication-ready visualizations and data tables, enabling the reproducible analysis of single cell experiments. It is planned to further extend WASP with additional analysis features and processing modules to meet future demands. As an example, the pre-processing of 10x data could be enhanced by optimized barcode error detection to increase the yield of barcodes separated from background noise. Furthermore, the integration of further single cell analysis platforms like BD Rhapsody is planned, and due to the modular design of WASP such extensions are easily possible.

## Availability and requirements

**Project name:** WASP - Web-based single cell RNA-Seq analysis platform

**Project home page:** http://www.computational.bio/software/wasp

**Repository:** https://github.com/andreashoek/wasp

**Operating system(s):** Pre-Processing: Linux; Post-Processing: Windows, Linux, MacOS

**Programming language:** R, Python/Snakemake

**Other requirements:** Pre-Processing: Linux, Docker or Conda, > 28 GB RAM; Post-Processing: web browser (e.g. Mozilla Firefox or Google Chrome), Standalone version on Windows, Docker on Linux or MacOS

**License:** GNU GPL version 3

**Any restrictions to use by non-academics:** None.

## Supplementary Information

**Additional file 1: Figure S1.** Schematic overview of the directory structure required for the WASP pre-processing workflow. The structure can be obtained from the git repository. Users only have to provide the reference genome FASTA file and the reference genome annotation GTF file in the Reference directory as well the raw FASTQ read files in the Samples directory.

**Additional file 2: Figure S2.** Screenshot of the summary.csv file. This shows used parameters during a WASP post-processing run, enabling to perform reproducible analyses.

## Data Availability

The ddSeq and 10x dataset used for assessing the pre- and post-processing are available at the NCBI Short Read Archive repository under the SRA accession number SRP149565 (ddSeq) and SRX4938158 (10x). Used reference genomes and annotations are available at the UCSC database under the version mm10 (ddSeq) and at the NCBI assembly database under the RefSeq accession number GCF_000224145.3 (10x). The 10x gene expression matrix used for testing the post-processing with externally generated datasets is available under https://support.10xgenomics.com/single-cell-gene-expression/datasets/1.1.0/pbmc3k

## Abbreviations

GUI: Graphical user interface
MDS: Multidimensional scaling
PCA: Principal component analysis
scRNA-Seq: Single cell RNA sequencing
SRA: Short Read Archive
t-SNE: t-Distributed Stochastic Neighbour Embedding
UMAP: Uniform Manifold Approximation and Projection
UMI: Unique molecular identifier
WASP: Web-accessible single cell RNA-Seq platform
