# Supplementary figures and images for: WASP: a versatile, web-accessible single cell RNA-Seq processing platform
Source: BMC Genomics. 2021 Mar 18;22:195. doi: 10.1186/s12864-021-07469-6 (PMC7977290; doi:10.1186/s12864-021-07469-6)

## Slide 1
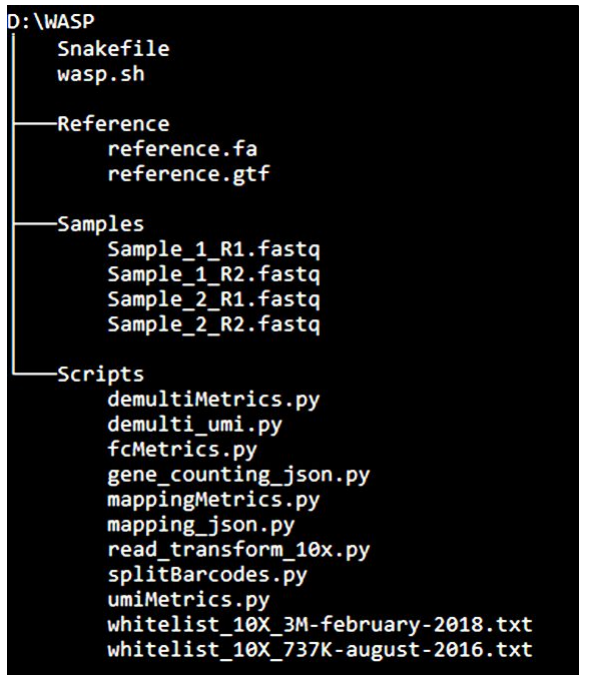

Supplement: Supplementary file 1 — Additional file 1: Figure S1. Schematic overview of the directory structure required for the WASP pre-processing workflow. The structure can be obtained from the git repository. Users only have to provide the reference genome FASTA file and the reference genome annotation GTF file in the Reference directory as well the raw FASTQ read files in the Samples directory. [file 12864_2021_7469_MOESM1_ESM.pptx]

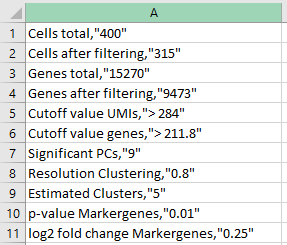

Supplement: Supplementary file 2 — Additional file 2: Figure S2. Screenshot of the summary.csv file. This shows used parameters during a WASP post-processing run, enabling to perform reproducible analyses. [file 12864_2021_7469_MOESM2_ESM.png]
